# Turkish Dentists’ Knowledge of Implant Options for Atrophic Jaws: A Cross-Sectional Survey

**DOI:** 10.7759/cureus.107514

**Published:** 2026-04-21

**Authors:** Furkan Aykut Erkurt, Mahmut Koparal, Halil Ibrahim Durmus, Ümit Solmaz

**Affiliations:** 1 Department of Oral and Maxillofacial Surgery, Faculty of Dentistry, Adıyaman University, Adıyaman, TUR

**Keywords:** all-on-4®, atrophic edentulous jaw, implantology knowledge, implant-supported rehabilitation, transnasal implant, zygomatic implant

## Abstract

Introduction: Atrophic edentulous jaws present significant challenges for implant rehabilitation. Although advanced techniques such as zygomatic, transnasal and All-on-4® (Nobel Biocare, Kloten, Switzerland) implants are increasingly used, the level of clinicians’ knowledge and clinical competence among dentists remains unclear. Therefore, this study aimed to evaluate the knowledge levels of Turkish dentists regarding advanced implant techniques in atrophic jaws and their self-reported clinical competencies.

Materials and methods: A cross-sectional online survey was conducted among 277 Turkish dentists using a structured 19-item questionnaire assessing demographics, knowledge, self-reported clinical competence, and educational expectations. Knowledge scores were categorized as low, moderate, or high. Associations between variables were analyzed using the chi-square test. The internal consistency of the Likert scale items was assessed using the Cronbach alpha coefficient.

Results: Among participants, 52.7% demonstrated moderate knowledge, while 27.8% showed high knowledge levels. Significant knowledge gaps were identified particularly for subperiosteal and zygomatic implants. Higher knowledge levels were significantly associated with greater preference for minimally invasive procedures, digital planning, and personalized treatment approaches (p < 0.05). The internal consistency of the proficiency scale was excellent (Cronbach's alpha = 0.912).

Conclusions: Turkish dentists demonstrate moderate knowledge but limited clinical confidence regarding advanced implant techniques. These findings highlight the need for improved integration of advanced implantology into undergraduate education and structured continuing professional development programs.

## Introduction

Severe alveolar bone resorption leading to atrophic jaws presents significant clinical challenges in current dental practice. A continuous remodeling process, beginning with tooth extraction, leads to progressive loss of alveolar bone, resulting in a deficiency in both vertical and horizontal directions [1]. The mandible is generally more affected by this resorption process than the maxilla, leading to anatomical limitations in conventional prosthetic methods [2].

As resorption progresses in edentulous jaws, both basic functions, such as mastication and speech, are negatively impacted, and facial aesthetics deteriorate. This often results in low self-esteem, social withdrawal, and impaired quality of life related to oral health [3]. Difficulty in achieving primary stability, the need for advanced augmentation techniques, or alternative implant designs are also among the challenges of low residual alveolar ridge height [4].

When atrophic jaws are rehabilitated, functional and psychological health is also improved, in addition to aesthetic improvement. Therefore, such cases require a multidisciplinary approach involving various specialties such as prosthodontics, oral and maxillofacial surgery, otorhinolaryngology, or plastic and reconstructive surgery [5]. Although bone augmentation procedures (e.g., sinus lift, onlay grafting) and advanced implant techniques (e.g., zygomatic, pterygoid, or transnasal implants) have increased treatment options, the successful application of these techniques by practitioners depends on their academic and surgical proficiency [6-8].

Recently, implant-supported fixed prostheses have been recognized as the gold standard prosthetic rehabilitation of atrophic edentulous jaws. However, treatment feasibility depends on patient-specific anatomic limitations, surgical expertise, and the availability of advanced techniques [9]. Offered as alternatives to extensive grafting procedures, the zygomatic implant, transnasal anchorage, and All-on-4® (Nobel Biocare, Kloten, Switzerland) protocol often allow immediate loading, reducing treatment time [10-12].

Despite the increasing prevalence and clinical importance of such advanced implant techniques, there is a lack of data on knowledge levels and practice patterns among dentists in specific geographic regions. Although implant dentistry is an integral part of postgraduate education and continuing professional education programs in Türkiye, the extent to which clinicians are competent with current surgical protocols applied in the treatment of atrophic edentulous jaws remains unclear.

Objective of the study

The primary objective of this study was to evaluate the knowledge level of Turkish dentists regarding advanced implant techniques in atrophic jaws. Secondary objectives included assessing self-reported clinical competence and identifying factors associated with knowledge levels. Our study also aims to provide insights for curriculum development.

## Materials and methods

Ethical approval

This study was approved by the Adıyaman University Non-interventional Clinical Research Ethics Committee at its meeting on March 19, 2024, under the number 2024/3-6.

Sample size and study population

A power analysis was performed using G*Power (Version 3.1.9.7, Heinrich-Heine-Universität Düsseldorf, Düsseldorf, Germany) software to determine the minimum required sample size. The effect size (f = 0.40) was determined according to Cohen's rules for a large effect size [13]. Given the exploratory nature of the study and the absence of directly comparable previous research evaluating dentists' knowledge of advanced implant techniques, a large effect size was assumed to ensure sufficient statistical power to detect meaningful differences between groups. Based on an effect size of 0.4, a significance level (α) of 0.05, and a power of 95%, the minimum number of participants required was calculated to be 180.

A total of 277 licensed dentists participated in the study. Inclusion criteria included dentists who graduated from a dental faculty in Türkiye and were actively practicing in any clinical setting, regardless of specialization.

Questionnaire design, validation, and distribution

The survey was developed following a comprehensive literature review on implant dentistry and survey-based studies evaluating dentists' knowledge levels and clinical decision-making processes. To the best of our knowledge, no previously validated survey specifically addressing advanced implant techniques in atrophic jaws has been identified. Therefore, a survey specific to the study was designed in accordance with the objectives of the current study. Content validity was evaluated by three experts in the field of oral and maxillofacial surgery, and minor changes were made to improve clarity and appropriateness. The internal consistency of the items on the Likert scale demonstrated excellent reliability (Cronbach's alpha = 0.912). The survey was distributed online via Google Forms (Google, Mountain View, CA, USA) using email and social media platforms (e.g., WhatsApp©, Meta Platforms Inc., Menlo Park, CA, USA). Data collection took place between March and December 2024. Participation in the survey was voluntary and did not require any personal information.

The questionnaire (see Appendix) consisted of 19 items divided into four sections: (i) Section 1: Demographic characteristics (4 items); (ii) Section 2: Knowledge-based multiple-choice questions (10 items); (iii) Section 3: Self-assessment of interventional competencies using a 5-point Likert scale; (iv) Section 4: Open-ended questions about future expectations, learning resources, and dental education.

Evaluation of knowledge level

Knowledge levels were calculated based on responses to 9 questions in Section 2 of the questionnaire: (i) Each correct answer was awarded 10 points;· (ii) Incorrect answers received 0 points.

The overall knowledge level was categorized as follows: (i) 0-30 points: Low knowledge level; (ii) 31-60 points: Moderate knowledge level; (iii) 61-90 points: High knowledge level.

The categorization of knowledge scores was adapted from previous survey-based studies in dental education literature.

Statistical analysis

Statistical analysis was performed using IBM SPSS Statistics version 22.0 (IBM Corp., Armonk, NY, USA). Categorical variables were expressed as frequencies and percentages (n, %). Continuous variables were assessed for normality using the Shapiro-Wilk test, if applicable. Since the variables in this study were predominantly categorical or ordinal in nature, statistical analyses were primarily conducted using non-parametric methods. Associations between categorical variables were evaluated using the chi-square test. Statistical significance was set at p < 0.05. The internal consistency of the Likert scale items assessing self-reported clinical competence was evaluated using the Cronbach’s alpha coefficient, which indicated excellent reliability (Cronbach’s alpha = 0.912).

## Results

A total of 277 dentists participated in the survey. In addition to demographic characteristics, the relationships between dentists’ workplace type, years of professional experience, participation in continuing education, and their knowledge levels and self-assessed competencies were analyzed.

Among participants, 112 (40.4%) were aged between 23 and 30 years, and female dentists accounted for 195 (70.4%) of the sample. The majority worked in private clinics or hospitals 179 (64.6%), followed by those employed at university hospitals 65 (23.5%) and public dental hospitals (28, 10.1%), and other institutions (5, 1.8%) (Table 1).

**Table 1:** Demographic characteristics of participants (n = 277) Data are presented as number (n) and percentage (%).

Variable	Category	n	%
Age	23–30	112	40.4
	31–40	80	28.9
	>40	85	30.7
Gender	Female	195	70.4
	Male	82	29.6
Workplace	Private clinic/hospital	179	64.6
	University hospital	65	23.5
	Public dental hospital	28	10.1
	Other	5	1.8

Analysis of responses to knowledge-based questions revealed that 77 (27.8%) of participants had a high level of knowledge, 146 (52.7%) had a moderate level, and 54 (19.5%) demonstrated a low level of knowledge (Table 2). The highest correct response rate, 268 (96.8%), was observed for the question on complications associated with mandibular nerve lateralization, while the lowest correct response rate, 134 (48.4%), was recorded for the question regarding the success rates of subperiosteal implants.

**Table 2:** Distribution of knowledge levels among participants Data are presented as number (n) and percentage (%).

Knowledge level	n	%
Low	54	19.5
Moderate	146	52.7
High	77	27.8

Chi-square analysis demonstrated no statistically significant association between gender and knowledge level (χ² = 2.331, df = 2, p = 0.312). Similarly, no statistically significant association was found between age group and knowledge level (χ² = 3.209, df = 6, p = 0.669).

There was also no statistically significant association between workplace setting and knowledge level (χ² = 4.070, df = 6, p = 0.544), indicating that knowledge distribution was relatively consistent across different clinical environments (Table 3).

**Table 3:** Association between knowledge level and demographic variables P-values were calculated using the Chi-square test. The Chi-square statistic (χ²) and degrees of freedom (df) are reported. Statistical significance was set at p < 0.05.

Variable	χ²	df	p-value
Gender	2.331	2	0.312
Age group	3.209	6	0.669
Workplace	4.070	6	0.544

Self-assessment results regarding interventional competencies indicated that the majority of participants lacked confidence in performing procedures involving pterygoid, transnasal, and subperiosteal implants. Chi-square analyses revealed no statistically significant association between knowledge level and clinical confidence in advanced implant procedures (p > 0.05) (Table 4).

**Table 4:** Association between dentists’ knowledge level and self-assessed competence in advanced implantology procedures Data are presented as number (n) and percentage (%). P-values were calculated using the Chi-square test. The Chi-square statistic (χ²) and degrees of freedom (df) are reported. Statistical significance was set at p < 0.05.

Procedure	Knowledge level low n (%)	Knowledge level moderate n (%)	Knowledge level high n (%)	χ²	df	p-value
All-on-4® implants	48 (17.3)	65 (23.5)	18 (6.5)	3.968	8	0.860
Zygomatic implants	71 (25.6)	100 (36.1)	25 (9.0)	4.883	8	0.770
Transnasal implants	113 (40.8)	102 (36.8)	25 (9.0)	4.821	8	0.777
Pterygoid implants	99 (35.7)	98 (35.4)	31 (11.2)	4.750	8	0.784
Subperiosteal implants	91 (32.9)	92 (33.2)	41 (14.8)	5.247	8	0.731
Short implants	75 (27.1)	109 (39.4)	71 (25.6)	4.516	8	0.808
Mandibular nerve lateralization	1 (0.4)	1 (0.4)	268 (96.8)	0.860	8	0.999
Buccolingually angled implants	113 (40.8)	164 (59.2)	77 (27.8)	10.707	8	0.219
Ramus graft procedures	67 (24.2)	59 (21.3)	139 (50.2)	2.254	8	0.972

The relationship between dentists’ knowledge levels and their preferences regarding future developments in implant-supported rehabilitation of atrophic jaws is presented in Table 5. 

**Table 5:** Relationship between dentists’ knowledge level and their preferences for future developments in implant-supported rehabilitation of atrophic jaws Data are presented as number (n) and percentage (%). P-values were calculated using the Chi-square test. The Chi-square statistic (χ²) and degrees of freedom (df) are reported. Statistical significance was set at p < 0.05.

Future development	Low n (%)	Moderate n (%)	High n (%)	Total n (%)	χ²	df	p-value
Minimally invasive procedures	35 (64.8)	111 (76.0)	70 (90.9)	216 (78.0)	13.270	2	0.001*
Faster healing times	27 (50.0)	96 (65.8)	62 (80.5)	185 (66.8)	13.477	2	0.001*
Higher success rates	34 (63.0)	102 (69.9)	66 (85.7)	202 (72.9)	9.944	2	0.007*
Lower-cost procedures	26 (48.1)	88 (60.3)	65 (84.4)	179 (64.6)	20.813	2	<0.001*
Personalized treatment plans	28 (51.9)	96 (65.8)	66 (85.7)	190 (68.6)	15.633	2	<0.001*
New techniques to reduce complications	32 (59.3)	114 (78.1)	64 (83.1)	210 (75.8)	10.674	2	0.005*

## Discussion

Our study provides important information about the knowledge levels and self-reported interventional competence of Turkish dentists regarding advanced implant techniques applied in atrophic edentulous jaws. Most participants showed a moderate level of knowledge. The predominance of female participants in this study may reflect the current demographic distribution of dentists in Türkiye, although sampling bias cannot be excluded. This finding is consistent with similar international studies showing that general dentists are unfamiliar with advanced techniques such as zygomatic and transnasal implants [14,15].

Results indicate that Turkish dentists mostly have a moderate level of knowledge about advanced implant techniques applied in atrophic edentulous jaws and lack confidence in their interventional competence. While awareness of techniques such as mandibular nerve lateralization, which has a long history in the literature, and the frequently applied All-on-4® concept is relatively high, there are knowledge gaps regarding zygomatic, pterygoid, and transnasal implants [8,16].

The high rate of correct answers to the mandibular nerve lateralization question indicates a good understanding of the underlying anatomical risks. However, low awareness regarding the success rates of subperiosteal or zygomatic implants highlights the need for current clinical training modules [17].

Participants with high knowledge scores showed greater interest in innovative technologies such as minimally invasive procedures, cone beam computed tomography (CBCT) assisted digital planning, and AI-assisted protocols compared to those with low scores. These findings are consistent with various studies in the literature highlighting that digital tools significantly improve diagnostic accuracy and increase clinician confidence in complex cases [18-20].

The similar knowledge levels of participants from diverse workplaces and age groups suggest that knowledge gaps are systemic rather than individual. This may stem from a lack of theoretical and practical training in advanced implant techniques during undergraduate education [21]. This finding may suggest potential gaps in education; however, causality cannot be established due to the cross-sectional design.

The strong interest in modern methods such as minimally invasive procedures and digital planning demonstrates a global trend toward evidence-based and individualized implantology. This is expected to facilitate the integration of technological advancements into clinical practice.

To meet the need for implant-supported rehabilitation in the geriatric population, advanced implant surgery techniques need to be included in undergraduate curricula and continuing education (CE) programs. The low self-confidence of dentists in advanced techniques such as pterygoid and transnasal implants suggests insufficient practical training from current undergraduate and continuing education programs. To address these shortcomings, modular certification programs, hands-on workshops, and clinical observation opportunities for advanced implantology are needed. Additionally, to ensure clinicians are prepared for the challenges of implant dentistry in practice, structured training modules on complex procedures should be promoted through national dental associations and specialist boards [22,23].

The reliance on self-reported data and the use of a non-random sampling method are limitations of this study. The use of convenience sampling via online platforms may have introduced selection bias. Self-reported competence may not reflect actual clinical performance. However, the wide geographic distribution of participants and their employment in different institutions increases the generalizability of the findings.

The findings of this study revealed a discrepancy between dentists' knowledge levels regarding advanced implant techniques and their self-reported clinical competence. While a significant proportion of participants possessed a moderate level of knowledge, limited confidence in applying techniques such as zygomatic and subperiosteal implants may influence clinical decision-making and referral patterns.

Improving clinicians' competence in advanced implant techniques can contribute to more predictable treatment planning and improved patient outcomes, particularly in the management of atrophic jaws.

Future research should focus on longitudinal and interventional study designs to assess the effectiveness of training programs in improving both knowledge and clinical competence. Additionally, to better understand the applicability of advanced implant techniques in the clinic, objective assessments of clinical competence are recommended, rather than clinicians' self-reported subjective measures.

## Conclusions

The study revealed that Turkish dentists possess a moderate level of knowledge regarding implant-supported rehabilitation for atrophic jaws. There is considerable interest in treatment approaches using minimally invasive procedures and current technology. To address the observed knowledge gaps, there is a need to integrate advanced implantology techniques into undergraduate curricula and to implement structured continuing education programs.

## Appendices

**Table 6:** Survey form

Informed consent
Dear Participant, this survey aims to evaluate dentists’ knowledge and clinical experience regarding implant-supported rehabilitation options in atrophic edentulous jaws. Participation is voluntary and anonymous. The survey consists of 19 questions and takes approximately 5–7 minutes. Data will be used solely for scientific purposes.
Principal Investigator: Research Assistant Furkan Aykut Erkurt, Adıyaman University Faculty of Dentistry, Department of Oral and Maxillofacial Surgery.
I agree to participate: ☐ Yes
1) Gender: ☐ Female ☐ Male
2) Age: ☐ 23–30 ☐ 31–40 ☐ 41–50 ☐ 51+
3) Year of graduation:
4) Current workplace: ☐ Private ☐ Public ☐ University ☐ Other:
5) All-on-4® characteristics: ☐ Reduced number of implants ☐ Angled posterior implants (30–45°) ☐ Immediate loading ☐ All of the above
6) Advantages of new generation subperiosteal implants: ☐ No graft needed ☐ Less invasive ☐ Faster healing ☐ All of the above
7) Success rate of new-generation subperiosteal implants: ☐ >90% ☐ 80–90% ☐ 70–80% ☐ <60%
8) Most common complication of zygomatic implants: ☐ Infraorbital paresthesia ☐ Sinus complications ☐ Bone necrosis ☐ Infection
9) Advantages of pterygoid implants: ☐ Reduce graft need ☐ High primary stability ☐ Posterior anchorage ☐ All of the above
10) Indications for transnasal implants: ☐ Atrophic mandible ☐ Atrophic maxilla ☐ Insufficient posterior maxillary bone ☐ B and C
11) Advantages of short implants (4–6 mm): ☐ Reduce graft need ☐ Less trauma ☐ Shorter treatment time ☐ All of the above
12) Most common complication after mandibular nerve lateralization: ☐ Infection ☐ Bleeding ☐ Esthetic issues ☐ Neurosensory disturbance
13) Opinion on buccal/lingual tilted implants in lateral atrophic mandible (multiple allowed): ☐ Beneficial in experienced hands ☐ Only selected patients ☐ Needs more evidence ☐ Alternatives preferred ☐ Do not use
14) Ramus graft harvesting site: ☐ Posterior mandibular ramus ☐ Retromolar region ☐ Iliac crest ☐ A and B
15) Self-reported competence (1=Strongly disagree, 5=Strongly agree): All-on-4®, Zygomatic implants, Transnasal implants, Pterygoid implants, Subperiosteal implants, Short implants, Mandibular nerve lateralization, Tilted implants, Ramus graft
16) Future developments (multiple allowed): ☐ Less graft need ☐ Less invasive surgery ☐ Faster healing ☐ Higher success ☐ Lower cost ☐ Personalized planning ☐ AI/robotics ☐ 3D printing ☐ Other
17) Sources of information (multiple allowed): ☐ Scientific articles ☐ Case reports ☐ Congresses/courses ☐ Online education ☐ Colleagues ☐ Other
18) Coverage during undergraduate education: ☐ Adequate ☐ Inadequate ☐ Not covered
19) Additional comments:

## References

[1] Hwaiti HS, Al-Rubidi YA, Al-Ashwal A, Al-Hadi Y, Al-Kibsi T, Al-Wesabi MA (2025). Inferior alveolar nerve relocation and immediate dental implant placement in severely resorbed mandibles: a prospective clinical case series. BMC Oral Health.

[2] Vaira LA, Biglio A, Van den Borre C, Salzano G, Lechien JR, Mommaerts MY, De Riu G (2026). Hybrid implant-supported rehabilitation of severely atrophic jaws using custom-made subperiosteal and conventional endosseous implants: a retrospective case series. J Craniomaxillofac Surg.

[3] Borges GA, Borges MH, Dini C, Marcello-Machado RM, Barão VA, Mesquita MF (2025). Prognosis of removable complete dentures considering the level of mandibular residual ridge resorption: a systematic review and meta-analysis. Clin Oral Investig.

[4] Vílchez B, Caneiro L, Lima C (2026). Clinical and radiographic performance of two distinct sandblasted, large-grit, acid-etched implant surfaces: a split-mouth randomized clinical trial. Clin Oral Implants Res.

[5] Chrcanovic BR, Albrektsson T, Wennerberg A (2016). Survival and complications of zygomatic implants: an updated systematic review. J Oral Maxillofac Surg.

[6] Aparicio C, Manresa C, Francisco K, Claros P, Alández J, González-Martín O, Albrektsson T (2014). Zygomatic implants: indications, techniques and outcomes, and the zygomatic success code. Periodontol 2000.

[7] Esposito M, Felice P, Worthington HV (2014). Interventions for replacing missing teeth: augmentation procedures of the maxillary sinus. Cochrane Database Syst Rev.

[8] Gutierrez de Pineres S, Omole D, Hirsch DL (2022). Rehabilitation of the severely atrophied maxilla: diverse indications and treatment protocols using zygomatic implants, standard implants, and bone grafting techniques. J Oral Maxillofac Surg.

[9] Davó R, Malevez C, Pons O (2013). Immediately loaded zygomatic implants: a 5-year prospective study. Eur J Oral Implantol.

[10] Maló P, Nobre Mde A, Lopes A, Ferro A, Moss S (2014). Five-year outcome of a retrospective cohort study on the rehabilitation of completely edentulous atrophic maxillae with immediately loaded zygomatic implants placed extra-maxillary. Eur J Oral Implantol.

[11] Alves MA, Both J, Mourão CF (2024). Long-term success of dental implants in atrophic maxillae: a 3-year case series using hydroxyapatite and L-PRF. Bioengineering (Basel).

[12] Soto-Penaloza D, Zaragozí-Alonso R, Penarrocha-Diago M, Penarrocha-Diago M (2017). The all-on-four treatment concept: Systematic review. J Clin Exp Dent.

[13] Jacob Cohen (1988). Statistical Power Analysis for the Behavioral Sciences, Second Edition. https://archive.org/details/statisticalpower0000cohe_j0l3/mode/2up.

[14] Aparicio C, Ouazzani W, Hatano N (2008). The use of zygomatic implants for prosthetic rehabilitation of the severely resorbed maxilla. Periodontol 2000.

[15] Laventure A, Lauwers L, Nicot R, Kyheng M, Ferri J, Raoul G (2022). Autogenous bone grafting with conventional implants vs zygomatic implants for atrophic maxillae: a retrospective study of the oral health-related quality of life. J Stomatol Oral Maxillofac Surg.

[16] Polido WD, Machado-Fernandez A, Lin WS, Aghaloo T (2023). Indications for zygomatic implants: a systematic review. Int J Implant Dent.

[17] Worthington P, Rubenstein J, Hatcher DC (2010). The role of cone-beam computed tomography in the planning and placement of implants. J Am Dent Assoc.

[18] Saini RS, Bavabeedu SS, Quadri SA (2024). Impact of 3D imaging techniques and virtual patients on the accuracy of planning and surgical placement of dental implants: a systematic review. Digit Health.

[19] Afrashtehfar KI, Abuzayeda MA, Murray CA (2024). Artificial intelligence in reconstructive implant dentistry—current perspectives. Prosthesis.

[20] Macrì M, D'Albis V, D'Albis G (2024). The role and applications of artificial intelligence in dental implant planning: a systematic review. Bioengineering (Basel).

[21] Andrews EA (2017). The future of interprofessional education and practice for dentists and dental education. J Dent Educ.

[22] Ferro AS, Nicholson K, Koka S (2019). Innovative trends in implant dentistry training and education: a narrative review. J Clin Med.

[23] Mattheos N, Wismeijer D, Shapira L (2014). Implant dentistry in postgraduate university education. Present conditions, potential, limitations and future trends. Eur J Dent Educ.

